# Supplementary material for: Analysis of treatment cost and persistence among migraineurs: A two-year retrospective cohort study in Pakistan
Source: PLoS One. 2021 Mar 26;16(3):e0248761. doi: 10.1371/journal.pone.0248761 (PMC7996986; doi:10.1371/journal.pone.0248761)
Supplement: S1 Table — (DOCX) [file pone.0248761.s001.docx]

| **No.** | **Private Hospitals** | | **Public Hospitals** | |
| --- | --- | --- | --- | --- |
|  | **Name** | **No of patients** | **Name** | **No of patients** |
| 1 | Liaquat national hospital | 190 | Jinnah post graduate medical center | 276 |
| 2 | Ziauddin hospital | 217 | Civil hospital | 198 |
| 3 | Taj medical hospital | 198 | Abbasi shaheed hospital | 201 |
| 4 | Anklesaria hospital | 179 | Sindh government hospital | 138 |

**S1 Table:** List of hospitals used for data collection

Total participants = 1597 N
