# Supplementary material for: Analysis of treatment cost and persistence among migraineurs: A two-year retrospective cohort study in Pakistan
Source: PLoS One. 2021 Mar 26;16(3):e0248761. doi: 10.1371/journal.pone.0248761 (PMC7996986; doi:10.1371/journal.pone.0248761)
Supplement: Attachment — Submitted filename: Response to Reviewers.docx [file pone.0248761.s004.docx]

**THE EDITORIAL OFFICE**	Dated	20-10-2020

**PLOS ONE**

Corrections in the manuscript entitled “ Analysis of treatment cost and persistence among migraineurs: A two-year retrospective cohort study in Pakistan (Ms. Ref. No.:  PONE-D-20-14782R2)”

Dear Editor,

First of all, authors are thankful to you for your useful comments and suggestions about our manuscript and giving us an opportunity to addressed reviewer’s comments with positive detailed corrections. The authors have modified the manuscript accordingly and the detailed corrections are listed below point by point.

**Response to Reviewer comments:**

**Reviewer # 1:**

**1. Thank you for giving me the opportunity to review the revised manuscript! In the response to reviewer comments, the authors stated that the data of patients with less than two-year follow-up period were now considered as censored data and removed from excluded patients lists. However, the hazard ratios in the result section did not change. This is confusing to me. If patients with less than two year follow-up were included in the updated analysis, the hazard ratios should be different from the original results.

Only including patients who completed 2-year follow-up in the analysis can cause significant bias. The follow-up should stop at the event date, loss of follow-up, or death, whichever is the earliest. In the current design, all patients who did not complete two-year follow-up were excluded. Even a minimum follow-up is needed to ensure sufficient follow-up time in some studies, the length of study period should not be used as the minimum follow-up period.**

**I would suggest the authors include patients who did not complete 2-year follow-up in the analysis and rerun the survival analysis.**

Response to Reviewer comment no. 1: The authors understand and agree with the reviewer points and concerns about study design especially for survival analysis (Hazards model and Kaplan Meier curve). Now after thorough review of literature related to these types of study and reviewer comments, authors included the data of all patients who completed minimum follow-up period of 12 months rather than 24 months to ensure the complete evaluation of 273 days continuation period including 30 days gap if occurs. In addition, the data of those patients who experienced the event (discontinuation) before the completion of minimum follow-up period were also included. However, the data of patients who drop-out during study due to any reason or didn’t experienced event during whole study period were censored in survival analysis (Hazards model and Kaplan Meier curve) as per reviewer recommendations.

***Data source***

*All cases of adult migraineur patients, irrespective of gender and ethnicity, who visited the hospitals were selected for this study on the following specific criteria including (a) patients who gave permission & were willing to join this study (b) a confirmed diagnosis of migraine and at least one episode of migraine during last one month and (c) all patients eligible for analysis who were followed up for minimum 12 months or the patients experienced the event at any date after first prescription filling.*

***Statistical analysis***

*Basically, the* cox *proportional hazard* regression model *is often used to* analyze *covariate information that changes over* time*, with the hazard proportional. Therefore we used the data of those patients who completed our minimum follow-up study period (1 year follow-up) or the event date for survival analysis while the data of patients who drop-out during study due to any reason or didn’t experienced event during whole study period were censored in survival analysis. In addition, without taking into account any possible confounding covariates, the cumulative proportion of patients persisting with their initial prescription drug of each study cohort were estimated using the Kaplan–Meier method along with log Rank test. All p-values are two-tailed with p<0.05 and p<0.0001 level of significance.*

**Table 6:** Statistical analysis of migraineurs persistence towards treatment using chi-square and multiple linear cox regression analysis

| **Risk factors** | **Cox Regression Analysis** | | |
| --- | --- | --- | --- |
|  | ***P-value*** | **HR^3^** | **CI^4^ (95%)** |
| **Migraineurs** | | | |
| Low Frequency | 0.0002 | – – |  |
| High frequency | <0.0001 | 1.628 | (1.221-2.179) |
| Chronic | 0.0002 | 0.881 | (0.881-0.912) |
| **Age** | <0.0001 | 1.293 | (1.092-1.458) |
| **Patients with depression** | <0.0001 | 1.268 | (1.084-1.458) |
| **Epilepticus seizures** | 0.0002 | 0.922 | (0.654-1.206) |
| **Patients with other comorbidities** | 0.0003 | 0.671 | (0.352-1.011) |
| **Class of drugs** | | | |
| NSAIDs | <0.0001 | – – |  |
| Combination analgesics | 0.0004 | 1.817 | (0.841-2.725) |
| Triptans | 0.0005 | 0.701 | (0.182-1.414) |
| Triptans + NSAIDs | <0.0001 | 0.758 | (0.501-1.289) |
| Prophylaxis drugs | <0.0001 | 1.314 | (0.958-1.424) |

^1^Continuers, ^2^Discontinuer, ^3^Hazard Ratio, ^4^Confidence interval, *p<0.0001*

*figures in parentheses indicate the reference category of categorical variables (hazard ratio=1), hazard ratio is adjusted for other variables in the table.

Data of patients who drop-out during the study period or didn’t experienced event during whole study period were censored in survival analysis

**Reviewer # 2:**

**1. The KM curve still does not look right. Though survival function can be estimated by linear interpolation in some special situation, KM curve (product limit estimator), which is a step function instead of a linear interpolated line, is preferred when there are censored patients. I recommend the authors check the example 4.1 from page 65 to 69 of the following book: Lee, Elisa T., and John Wang. Statistical methods for survival data analysis. Vol. 476. John Wiley & Sons, 2003.**

Response to Reviewer comment no. 1: First of all authors are highly thankful to reviewer for shared us the previous literature for the preparation of KM curve. Now after thorough review of literature related to these types of study and reviewer provided book, KM curve has been plotted correctly after including censored data as well. In addition the result of Log Rank test also applied to evaluate the significant difference in treatment persistence among users of different anti-migraine therapy.

*Data of patients who drop-out during study due to any reason or didn’t experienced event during whole study period were censored

**Figure 1: Kaplan–Meier curves showing adjusted cumulative probability of treatment persistence for 24 months after by anti-migraine drugs**Top of Form

1. Bottom of Form
