# Supplementary material for: Analysis of treatment cost and persistence among migraineurs: A two-year retrospective cohort study in Pakistan
Source: PLoS One. 2021 Mar 26;16(3):e0248761. doi: 10.1371/journal.pone.0248761 (PMC7996986; doi:10.1371/journal.pone.0248761)
Supplement: Attachment — Submitted filename: Response to Reviewers.docx [file pone.0248761.s005.docx]

**THE EDITORIAL OFFICE**	Dated	15-01-2021

**PLOS ONE**

Corrections in the manuscript entitled “Analysis of treatment cost and persistence among migraineurs: A two-year retrospective cohort study in Pakistan (Ms. Ref. No.:  PONE-D-20-33485)”

Dear Editor,

First of all, authors are thankful to you for your useful comments and suggestions about our manuscript and giving us an opportunity to addressed reviewer’s comments with positive detailed corrections. The authors have modified the manuscript accordingly and the detailed corrections are listed below point by point.

**Response to Reviewer comments:**

**Reviewer # 1:**

**1. Please clarify the reference level for HR of non-binary variables in the abstract.**

Response to Reviewer comment no. 1: The reference level for hazard ratios of non-binary variables has been clarified in the abstract.

***Abstract***

*Pairwise comparisons from Cox regression/hazards ratio were used to assess the predictors of persistence with the reference category of non-binary variables i.e. hazard ratio = 1 for low frequency migraineurs and NSAIDs users.*

**2. The font of number in table 5 is inconsistent with the font of number in other tables.**

Response to Reviewer comment no. 2: The font size has been corrected.

**3. In the data source section, the authors mentioned that “all cases of adult migraineur patients, irrespective of gender and ethnicity, who visited the hospitals were selected for this study on the following specific criteria including … (c) all patients eligible for analysis were followed up for minimum 12 months”. The time in the study should not be one of the eligibility criteria for the survival analysis as patients who dropped out could still provide information about the event of interest by right censoring.**

Response to Reviewer comment no. 3: We authors are fully agreed with the reviewer concern about this point. Basically this criterion was added by the recommendation of previous reviewer in his first round of review. However, in this study minimum 12 months follow-up criteria was only consider for the determination of patient persistence (273 days continuation) with migraine treatment while all patients data which were initially enrolled in our study (2043 N) including dropped out patients during our study period were also already included during survival analysis as indicated in Kaplan Meier curves and HR model.

In addition, correction has been made for this specific criterion for further clarification.

Similarly, it has already been mentioned in statistical analysis that the dropped out patients were also included in survival analysis as right censored data.

The sentence has also been added in abstract as well.

***Data source***

*All cases of adult migraineur patients, irrespective of gender and ethnicity, who visited the hospitals, were selected for this study on the following specific criteria including (a) patients who gave permission & were willing to join this study and (b) a confirmed diagnosis of migraine and at least one episode of migraine during last one month. However, patients who were completed the minimum followed up period of 12 months or had an experienced of any event (discontinuation or switches) during this period were included for the determination of treatment persistence i.e. 273 days continuation.*

***Statistical analysis***

*The* Cox *proportional hazard* regression model *is often used to* analyze *covariate information that changes over* time*, with the hazard proportional. Therefore we used the data of all study patients including the data of patients who drop-out during study due to any reason as right censored data in survival analysis.*

***Abstract***

*The minimum follow up period for each migraineur was about 12 months for persistence analysis while dropped-out patients data were also included in survival analysis as right censored data.*

**4. In the statistical analysis section, please clarify the meaning of the sentence “Therefore we used the data of those patients who completed our minimum follow-up study period (1 year follow-up) or the event data for survival analysis …”.**

Response to Reviewer comment no. 4: This sentence was also added by the recommendation of previous reviewer in his first round of review. Now sentence has been modified and more clarified as per reviewer recommendation.

***Statistical analysis***

*Therefore we used the data of all study patients including the data of patients who drop-out during study due to any reason as right censored data in survival analysis.*

**Reviewer # 2:**

**1. In the method section, the authors stated that they used the data of those patients who completed our minimum follow-up study period (1 year follow-up) or the event date for survival analysis while the data of patients who drop-out during study due to any reason or didn’t experienced event during whole study period were censored in survival analysis. This description is a bit confusing to me. If the minimum follow-up study period is an inclusion criterion, patients with less than 1-year follow-up would be excluded. Please consider clarifying the patients included in the analysis.**

Response to Reviewer comment no. 1: Similar concern is also raised by first reviewer. We authors are fully agreed with the reviewers concern about this point. Basically this criterion was added by the recommendation of previous reviewer in his first round of review. However, in this study minimum 12 months a follow-up criterion was consider for the determination of patient persistence (273 days continuation) with migraine treatment. Therefore, sample size had already been reduced to 1597 N from 2043 N in all initial tables. However, all patients’ data which were initially enrolled in our study (2043 N) including dropped out patients during our study period were also included during survival analysis as indicated in Kaplan Meier curves and HR model.

In addition, correction has been made for this specific criterion for further clarification.

Similarly, it has already been mentioned in statistical analysis that the dropped out patients were also included in survival analysis as right censored data.

The sentence has also been added in abstract as well.

***Data source***

*All cases of adult migraineur patients, irrespective of gender and ethnicity, who visited the hospitals, were selected for this study on the following specific criteria including (a) patients who gave permission & were willing to join this study and (b) a confirmed diagnosis of migraine and at least one episode of migraine during last one month. However, patients who were completed the followed up period of 12 months were included for the determination of treatment persistence i.e. 273 days continuation.*

***Statistical analysis***

*The* Cox *proportional hazard* regression model *is often used to* analyze *covariate information that changes over* time*, with the hazard proportional. Therefore we used the data of all study patients including the data of patients who drop-out during study due to any reason as right censored data in survival analysis.*

***Abstract***

*The minimum follow up period for each migraineur was about 12 months for persistence analysis while dropped-out patients data were also included in survival analysis as right censored data.*

**2. Also, some terms used in the manuscript are still confusing to me. For example, the title of table 6 is "Statistical analysis of migraineurs adherence towards treatment using chi-square and multiple linear cox regression analysis". Although a proportional Cox regression model can be converted to a linear regression model, the term of multiple linear cox regression analysis is confusing because I believe a proportional hazards model is used in the study and the model was not converted to a linear model.**

Response to Reviewer comment no. 2: Authors really thankful to reviewer for clarifying this point. The term has been corrected as per reviewer recommendation.

***Table 6:*** *Statistical analysis of migraineurs persistence towards treatment using chi-square and cox proportional hazard regression analysis*

**3. In addition, I found a few grammar mistakes. For example, in Line 21 on page 17, "one of the study concluded" should be "one study concluded" or "one of the studies concluded". Similar grammar mistakes can be found elsewhere in the manuscript.**

Response to Reviewer comment no. 3: All grammar mistakes have been corrected now.

*However, persistence with prevalent patients showed different results in comparison with incident patients and approximate 70% of prevalent patients exhibit non-persistence behavior with their treatment which could be due to avoid some potential hazards associated with drug and one study concluded the same results of poor persistence with prevalent / prophylactic treatment [9].*

**4. Please also consider changing all p-values that are 0.000 to p<0.001. Theoretically, p-values cannot be equal to zero.**

Response to Reviewer comment no. 4: All p-values have been corrected as per reviewer recommendation.
