# Supplementary material for: Analysis of treatment cost and persistence among migraineurs: A two-year retrospective cohort study in Pakistan
Source: PLoS One. 2021 Mar 26;16(3):e0248761. doi: 10.1371/journal.pone.0248761 (PMC7996986; doi:10.1371/journal.pone.0248761)
Supplement: Attachment — Submitted filename: Response to Reviewers.docx [file pone.0248761.s006.docx]

**THE EDITORIAL OFFICE**	Dated	03-03-2021

**PLOS ONE**

Corrections in the manuscript entitled “Analysis of treatment cost and persistence among migraineurs: A two-year retrospective cohort study in Pakistan (Ms. Ref. No.:  PONE-D-20-33485R1)”

Dear Editor,

First of all, authors are thankful to you for your useful comments and suggestions about our manuscript and giving us an opportunity to addressed reviewer’s comments. The authors have modified the manuscript accordingly and the detailed corrections are listed below point by point.

**Response to Reviewer comments:**

**Reviewer # 1:**

**1. Abstract (do not indent; must include OBJECTIVES, METHODS, RESULTS, CONCLUSION)**

Response to Reviewer comment no. 1: Abstract has been structured as per reviewer recommendation.

***Abstract***

***OBJECTIVES:*** *The persistence pattern of anti-migraine drugs’ use among migraineurs is very low in the United States and different European countries. However, the cost and persistence of antimigraine drugs in Asian countries have not been well-studied. Hence, the present study aimed to evaluate the treatment cost and persistence among migraineurs in Pakistan.* ***METHODS:*** *Data from prescriptions collected from migraineurs who visited the Outpatient Department (OPD) of different public and private sector tertiary-care hospitals of Karachi, Pakistan were used to conduct this retrospective cohort study from 2017 to 2019. The minimum follow up period for each migraineur was about 12 months for persistence analysis while dropped-out patients data were also included in survival analysis as right censored data. Pairwise comparisons from Cox regression/hazards ratio were used to assess the predictors of persistence with the reference category of non-binary variables i.e. hazard ratio = 1 for low frequency migraineurs and NSAIDs users. Persistence with anti-migraine drugs was estimated using the Kaplan-Meier curve along with the Log Rank test.* ***RESULTS:*** *A total of 1597 patients were included in this study, 729 (45.6%) were male and 868 (54.3%) were female. Non-steroidal anti-inflammatory drugs (NSAIDs) were the most prescribed class of drug initially for all classes of migraineurs (26.1 %). Of them, 57.3% of migraineurs discontinued their treatment, 28.5% continued while 14.8% were switched to other treatment approaches. Persistence with initial treatment was more profound in female (58.8%) patients compared to males while the median age of continuers was 31 years. The total cost of migraine treatment in the entire study cohort was 297532.5 Pakistani Rupees ($1901.1). By estimating the hazard ratios (HR) using the cox regression analysis, it can be observed that patients with high frequency (HR, 1.628; 95%CI, 1.221-2.179; p<0.0001) migraine, depression (HR, 1.268; 95%CI, 1.084-1.458; p<0.0001), increasing age (HR, 1.293; 95%CI, 1.092-1.458; p<0.0001), combination analgesics (HR, 1.817; 95%CI, 0.841-2.725; p=0.0004) and prophylaxis drugs (HR, 1.314; 95%CI, 0.958-1.424; p<0.0001) users were at a higher risk of treatment discontinuation. However, patients with chronic migraine (HR, 0.881; 95%CI, 0.762-0.912; p=0.0002), epileptic seizure (HR, 0.922; 95%CI, 0.654-1.206; p=0.0002), other comorbidities (HR, 0.671; 95%CI, 0.352-1.011; p=0.0003) and users of triptan(s) (HR, 0.701; 95%CI, 0.182-1.414; p=0.0005) and triptan(s) with NSAIDs (HR, 0.758; 95%CI, 0.501-1.289; p<0.0001) had more chances to continue their initial therapy.* ***CONCLUSION:*** *Similar to western countries, the majority of migraineurs exhibited poor persistence to migraine treatments. Various factors of improved persistence were identified in this study.*

**2. -In abstract, please rewrite sentence "..1597 “N” migraineurs...".**

Response to Reviewer comment no. 2: The sentence has been corrected.

*A total of 1597 patients were included in this study, 729 (45.6%) were male and 868 (54.3%) were female.*
